# Early escalation of oral lipid-lowering therapy to achieve LDL-cholesterol targets in patients after myocardial infarction: study protocol for the prospective, open-label HAnnover ChOlesterol Lowering in Acute Coronary Syndrome trial

**DOI:** 10.3389/fcvm.2026.1755590

**Published:** 2026-06-03

**Authors:** Andreas Schäfer, Anika Großhennig, Leonie Theis, Christoph Schindler, Esther Grahl, Dirk O. Stichtenoth, Tobias König, Welf-Axel Geller, Hannes Hermann Jakob Günter Schmidt, Udo Bavendiek, Johann Bauersachs

**Affiliations:** 1 Department of Cardiology and Angiology, Hannover Medical School, Hannover, Germany; 2 Institute of Biostatistics, Hannover Medical School, Hannover, Germany; 3 Center for Clinical Trials, Hannover Medical School, Hannover, Germany; 4 Department of Clinical Pharmacology, Hannover Medical School, Hannover, Germany

**Keywords:** acute coronary syndrome, bempedoic acid, ezetemibe, LDL - cholesterol, lipid lowering

## Abstract

**Background:**

Contemporary registry data indicate that only 20%–25% of patients following acute myocardial infarction achieve guideline-recommended low-density lipoprotein cholesterol (LDL-C) values <1.4 mmol/L (55 mg/dL). The present study aims to evaluate the impact of early intensive therapy (potent statin + ezetimibe ± bempedoic acid) on lipid control after myocardial infarction.

**Methods:**

This is a prospective, interventional, single-center study designed to assess the effectiveness of bempedoic acid in achieving LDL-C targets in patients following ST-elevation or non-ST-elevation myocardial infarction who remain insufficiently treated with atorvastatin plus ezetimibe. Patients not previously treated with lipid-lowering agents and presenting with admission LDL-C > 2.6 mmol/L (100 mg/dL) will be treated with atorvastatin (at least 40 mg/day or equivalent) plus ezetimibe (10 mg/day) for 6 weeks. Thereafter, the subgroup of patients failing to reach an LDL-C < 1.4 mmol/L (55 mg/dL) will be escalated to additive bempedoic acid (180 mg/day) for another 8 weeks. Those who reach the target will continue the aforementioned therapy. The primary endpoint is the proportion of patients who successfully achieve an LDL-C < 1.4 mmol/L (55 mg/dL) after 8 weeks of treatment with triple therapy. Secondary endpoints include the proportion of patients successfully reaching the target in the overall population and in the background medication group alone.

**Discussion:**

The results of this study will provide novel insights into post-infarction LDL-C control by evaluating the usefulness of an early intensive escalation oral lipid-lowering treatment strategy. Early intensive lipid-lowering triple oral therapy may facilitate the achievement of guideline-recommended LDL-C levels within the first 3 months after myocardial infarction compared with current clinical practice.

**Clinical Trial Registration:**

EudraCT 2022-003526-50

## Background

Low-density lipoprotein cholesterol (LDL-C) is one of the most important modifiable cardiovascular risk factors (1). In 2019, European guidelines lowered the recommended target LDL-C from <1.8 mmol/L (70 mg/dL) to <1.4 mmol/L (55 mg/dL) for secondary prevention in coronary artery disease (2). Despite well-intended recommendations and strong evidence, the previous LDL-C target of <1.8 mmol/L (70 mg/dL) had only been achieved in 21% in of patients included in registries (3, 4). In Germany, the reported proportion was even lower, with ≤15% of patients achieving the target following an acute coronary syndrome (5, 6).

After broader availability of more potent statins such as atorvastatin and rosuvastatin, we recently assessed the achievement of LDL-C guideline targets in real-world everyday clinical practice postmyocardial infarction. In brief, early standardized potent lipid-lowering therapy with atorvastatin plus ezetimibe resulted in nearly every second patient discharged after ST-elevation myocardial infarction (STEMI) achieving an LDL-C < 1.4 mmol/L (55 mg/dL) (7), with even higher rates in those receiving high-dose potent statins combined with ezetimibe (8).

In addition to high-intensity potent statins plus ezetimibe, proprotein convertase subtilisin/kexin type 9 (PCSK9) inhibitors such as evolocumab or alirocumab have shown additive clinical benefit in cardiovascular outcome trials (9, 10), but are not widely available for the overall population due to prescription limitations. In Germany, oral combination therapy must be escalated to the maximum level with documented inefficiency to reach the guideline-recommended LDL-C target prior to prescription of PCSK9 inhibitors. In the meantime, bempedoic acid has demonstrated a significant reduction in cardiovascular events when used as an oral add-on therapy in case of statin intolerance (11). Beyond that, adding bempedoic acid to maximally tolerated statins in high-cardiovascular-risk patients results in a further reduction of LDL-C by 17% (12). Therefore, bempedoic acid may offer a potential option to achieve guideline-recommended LDL-C targets <1.4 mmol/L (55 mg/dL) when standard combinations of a potent statin and ezetimibe are insufficient, and is currently required as part of stepwise escalation treatment prior to PCSK9 inhibition.

Several real-world registries document low achievement rates for guideline-recommended targets, with only one out of five patients reaching LDL-C < 1.4 mmol/L (55 mg/dL) (13, 14). However, recent prospective observations documented achievement rates of almost 50% with high-dose potent statin combined with ezetimibe (15), consistent with our own retrospective findings (7). Early combination of highly potent statin therapy with ezetimibe and bempedoic acid can further improve the response rate, as demonstrated in the LAI-REACT trial (16), though the ES-BempedACS trial showed no additional benefit (15). In that trial, however, one-third of patients were already receiving lipid-lowering therapy (15).

We planned and initiated the HAnnover ChOlesterol Lowering in Acute Coronary Syndrome (HACOL-ACS) trial to address three major clinically relevant questions: First, how many patients after a myocardial infarction reach the guideline-recommended target [LDL-C < 1.4 mmol/L (55 mg/dL)] on dual therapy with atorvastatin (at least 40 mg/day or equivalent) plus ezetimibe (10 mg/day)? Second, how many of the patients not achieving the target on a dual statin/ezetimibe therapy reach the target after adding bempedoic acid? Third, how many patients miss the target despite escalated oral triple lipid-lowering therapy and therefore need referral for PCSK9 inhibition? Our trial is distinct from the ES-BempedACS trial, as it focuses exclusively on patients without previous lipid-lowering therapy and will demonstrate the exact additive impact of bempedoic acid on top of dual therapy in each patient due to its sequential escalation strategy. Thereby, we will evaluate the real-world, guideline-mandated stepwise oral escalation therapy and quantify the residual unmet need for LDL-C target achievement. Starting with dual background medication is consistent with current guideline recommendations (17), and sequential add-on with bempedoic acid is also guideline-recommended (17), although this approach has never been tested in clinical practice. It is therefore important to document the efficacy of additive bempedoic acid on top of established and tolerated high-intensity statin treatment in combination with ezetimibe, to provide data supporting such recommendations. This is particularly important for the trial to be conducted in a sequential and clear manner as recent data from the ES-BempedACS trial with varying pretreatment populations did not show an additive LDL-C-lowering effect of bempedoic acid (15). Similarly, data from a recent oral PCSK9 inhibitor trial showed only a marginal additive effect in the bempedoic acid-treated comparator group when added to stable statin therapy (18).

The results of HACOL-ACS will also provide meaningful information for patients beyond the German healthcare system. While the trial design is adapted to national settings, the proportion of patients achieving guideline-recommended LDL-C targets at the end of the trial with double and triple therapies will be applicable both in regions with sequential dose escalations and in those allowing triple therapy from the beginning.

## Methods/design

Reporting guidelines from Standard Protocol Items: Recommendations for Interventional Trials (SPIRIT) were used for this manuscript and table 2 (19). The HACOL-ACS trial is registered in the EudraCT database (Identification number: 2022-003526-50), which collects all items required by the World Health Organization Trial Registration Data Set. The trial has been approved by the local ethics committee at Hannover Medical School. Coordination is provided by the Center for Clinical Trials, including administrative tasks and data management. Patients undergo study-related procedures at the trial site (Department of Cardiology at Hannover Medical School, Germany). Statistic planning and evaluation are performed by the Institute of Biostatistics, and pharmacovigilance is overseen by the Department of Clinical Pharmacology.

### Study design

**Figure 1:**
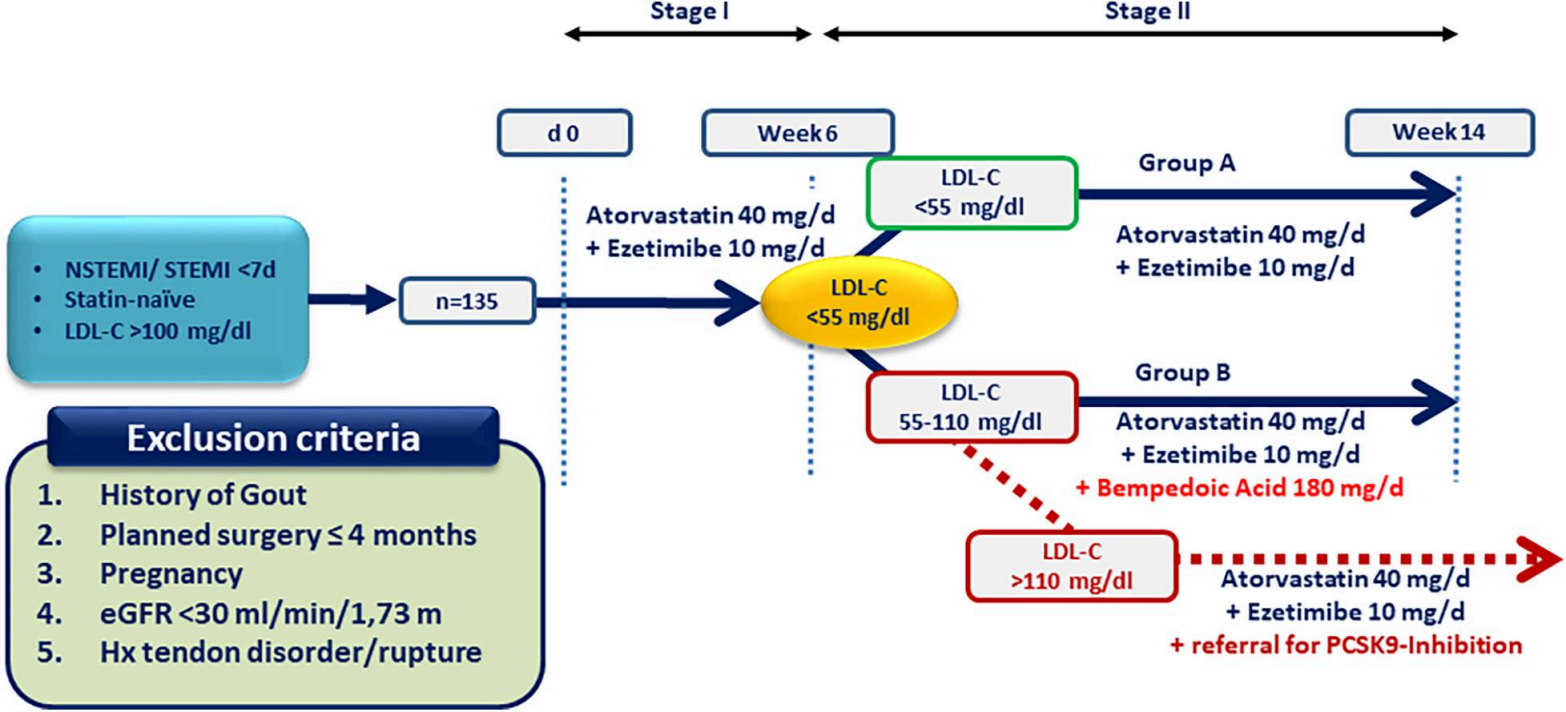
Study algorithm. eGFR, estimated glomerular filtration rate; Hx, History of …; LDL-C, low-density lipoprotein-cholesterol; NSTEMI, Non-ST-segment myocardial infarction; STEMI, ST-segment myocardial infarction.

The study design according to protocol version 3.0 dated 13 November 2023, is depicted in Figure 1. This is a single-center, open-label, prospective, interventional study designed to precisely estimate the proportion of patients who successfully achieve LDL-C treatment targets as recommended by current guidelines. In Stage I, up to 135 patients will treated for 6 weeks with atorvastatin (at least 40 mg/day or equivalent) plus ezetimibe (10 mg/day). The chosen statin and its dose reflect the recommended regional background medication, established by consensus among local PCI hospitals, the regional Association of Statutory Health Insurance Physicians, and regional representatives for outpatient cardiologists. This consensus balances additive LDL-C-lowering efficacy against potential side effects (7). Thereafter, in Stage II, patients are split into two groups:

Group A will consist of the subgroup of patients who do not reach the LDL-C guideline target [LDL-C < 1.4 mmol/L (55 mg/dL)] after 6 weeks and will receive additive bempedoic acid (180 mg/day) for another 8 weeks (Triple therapy). Group B will consist of the subgroup of patients who achieve the LDL-C guideline target after 6 weeks of treatment with atorvastatin plus ezetimibe and who will continue on dual therapy for another 8 weeks (Dual therapy).

The 6-week escalation interval reflects local healthcare system, including certain reimbursement constraints, while aligning with evidence-based preferences to minimize delays in treatment intensification.

### Study population and inclusion

The study population will comprise up to 135 patients aged ≥18 and ≤85 years following acute percutaneous coronary intervention for STEMI or non-STEMI (NSTEMI), all on high-intensity oral lipid-lowering therapy. Importantly, baseline parameters will be used to demonstrate that patients enrolled in the HACOL-ACS trial are representative of a real-world cohort described previously at our institution, which showed mean LDL-C levels of 3.9 ± 0.7 mmol/L (149 ± 27 mg/dL) (7).

Inclusion criteria for the trial are as follows: patients of all sexes aged between 18 and 85 years, who have provided written informed consent, had NSTEMI or STEMI with successful coronary intervention within 7 days prior to screening, have a therapy-naïve LDL-C > 2.6 mmol/L (100 mg/dL), and are able to cooperate with the protocol regimen and follow-up.

Exclusion criteria are as follows: history of gout, scheduled surgery within the next 4 months, inability to attend study site revisits, participation in another clinical trial within 30 days prior to study start or during the trial, known hypersensitivity to any of the components of the medications used, pregnancy or breastfeeding, severe renal disorders (defined as estimated glomerular filtration rate <30 mL/min/1.73 m^2^) or requiring dialysis for end-stage renal disease, history of tendon disorders or tendon rupture, or requirement for placement in a mental institution by court or official order.

#### Study intervention/treatment

After the index hospitalization and enrolment into the trial, standard medication including atorvastatin (40 mg/day) and ezetimibe (10 mg/day) will be recommended. After 6 weeks (Stage I), a study visit will be conducted at the institution and another lipid test will be performed. Next (Stage II), study medication (bempedoic acid, 180 mg/day, provided by Daiichi Sankyo, Munich, Germany) will be given at V2 to patients with LDL-C 1.4-2.8 mmol/L (55-110 mg/dL) (Group A), and patients with LDL-C < 1.4 mmol/L (55 mg/dL) will be continued on the background medication with the previous dosing (Group B). Patients with LDL-C > 2.8 mmol/L (110 mg/dL) at week 6 will be directly referred for PCSK9 inhibition outside the trial. Patients with LDL-C ≤ 2.8 mmol/L (110 mg/dL) mg/dL will have a revisit (V3) after another 8 weeks, including another lipid test.

The dose of bempedoic acid— 180 mg/day—will not be modified. The background medication is atorvastatin (40 mg/day) plus ezetimibe (10 mg/day), which is the standard discharge medication. If—despite explanation about the study protocol—patients are escalated by their treating physicians to higher doses of atorvastatin (>40 mg/day) or switched to rosuvastatin (minimum 20 mg/day), this will be considered stable baseline medication with regard to the study protocol. Switching between medications and de-escalation of statin treatment are strongly discouraged. A sensitivity analysis excluding patients with escalated doses of atorvastatin or switching to equal doses of rosuvastatin is planned.

Remaining study medication will be counted upon return at V3. Aside from adherence to lipid-lowering therapy, no other restrictions apply to routine care.

This strategy reflects current guideline recommendations to either initiate or escalate to a dual lipid-lowering therapy with a high-dose potent statin in combination with ezetimibe prior to adding a third compound (17). It also supports the generalizability of HACOL-ACS results to broader patient populations.

#### Study endpoints

The primary endpoint is defined as the proportion of patients (%) in Group A who successfully achieve the European Society of Cardiology (ESC) LDL-C guideline target after Stage II. Secondary endpoints comprise the following: proportion of patients (%) who successfully achieve ESC LDL-C guideline targets [LDL-C < 1.4 mmol/L (55 mg/dL)] after treatment with dual therapy including atorvastatin (at least 40 mg/day or equivalent) plus ezetimibe (10 mg/day) at 6 and at 14 weeks; the proportion of patients (%) who successfully achieve the ESC LDL-C guideline targets after 14 weeks of treatment; the proportion of patients (%) who achieve American heart Association (AHA)/American College of Cardiology (ACC) guideline-recommended treatment targets of LDL-C < 1.8 mmol/L (70 mg/dL) after 14 weeks of treatment in the triple therapy group; mean change from baseline to week 6 and week 14 in LDL-C, total cholesterol, HDL-C, triglycerides, uric acid, creatine kinase, systolic and diastolic blood pressure, and heart rate; the proportion of non-compliant patients (%) taking less than 90% of the allocated study medication; and mean change from baseline to week 6 and to week 14 in quality of life.

Safety will be assessed by monitoring and recording all adverse events, serious adverse events, and certain laboratory parameters (further described under pharmacovigilance).

The primary endpoint was chosen to focus on the efficacy of the investigational compound in order to prevent that a higher than anticipated achievement rate of the LDL-C target on the baseline medication might result in an escalation subgroup being too small to conduct proper statistical analyses.

#### Follow-up

Treatment on-site visits will be performed at V2 (Week 6 ±5 days) and V3 (Week 14 ±5 days). An additional safety follow-up visit will be conducted by telephone at week 15 (±5 days). The assessments performed at each visit are summarized in Table 1. Adherence to background medication will be assessed by interview, and adherence to bempedoic acid will be assessed by pill count.

**Table 1:** Study calendar.

Visit	Screening	Baseline	Treatment visits		Safety follow-up visit (by phone)
		V1	V2	V3^a^	V4^b^
Time	1–14 days prior to baseline	Day 0	Week 6 (±5 days)	Week 14 (±5 days)	Week 15 (±5 days)
Informed consent	x				
Inclusion/exclusion criteria	x				
Demographic data	x				
Medical history	x				
Physical examination/vital signs		x	x	x	
Quality of life questionnaire (SF-36)		x	x	x	
Lipid profile (including total cholesterol, HDL-C, LDL-C, and triglycerides)	x^c^		x	x	
Uric acid, creatine kinase	x^c^		x	x	
Dispense of study medication			X^d^		
Liver function test			x	x	
Serum *β*-hCG pregnancy test (patients of childbearing potential)	x		x	x	
Concomitant medication	x	x	x	x	
Adverse events				x^e^	x^e^
Return of IMP				x	

AEs, adverse events.

a Patients with LDL-C ≤ 2.8 mmol/L (110 mg/dL) will have a revisit (V3) 8 weeks after V2.

b Telephone inquiry regarding AEs after IMP discontinuation (triple therapy group) and equivalent time point for dual therapy group.

c Admission laboratory values from routine testing may be included within the screening period.

d Study medication will be given to patients with LDL-C ≥ 1.4 mmol/L (55 mg/dL). Patients with LDL-C > 2.8 mmol/L (110 mg/dL) will be directly recommended for PCSK9 inhibition.

e Triple therapy group and double therapy group from dispensation of IMP onward (V2).

All trial data will be entered into an Electronic Case Report Form provided by the Center for Clinical Trials, which will also conduct the monitoring.

#### Sample size

The number of patients for this study is based on previous experiences in secondary prevention surveys conducted at our institution (7). The primary objective of this study is to precisely estimate the proportion of patients who reach the ESC LDL-C guideline target of LDL-C < 1.4 mmol/L (55 mg/dL) after treatment Stage II in the triple therapy group. A sample size of *n* = 135 will enable us to estimate the responder rate with an accuracy of at least ±13.2%, corresponding to a confidence interval width of 26.4%. It is assumed that the responder rate is between *p* = 0.3 and *p* = 0.5. Since the standard deviation is maximal at *p* = 0.5, the confidence interval is the widest at *p* = 0.5. Hence, we assume a responder rate of 0.5 for a conservative sample size calculation. A small drop-out rate of 5% is expected in post-myocardial infarction patients. The primary analysis will be carried out in the intention-to-treat (ITT) population. For interpretation of the study results, analysis of the per-protocol (PP) population is also of clinical importance. To draw robust conclusions, consistency between ITT and PP results is needed. To account for potential drop-outs, we compute the sample size for the PP population. To determine an exact two-sided 95% Clopper–Pearson confidence interval with a width of 26.4%, assuming a responder rate *p* = 0.5, a sample size of 60 patients in the triple therapy group is needed.

Based on previous studies (7), approximately half of the enrolled patients will not achieve the goal of LDL-C < 1.4 mmol/L (55 mg/dL) during Stage 1. Because this is only an estimation and a drop-out rate of 5% is assumed, we will include up to 15 additional patients. This results in a total sample size of maximum 2*60 + 15 = 135 patients for the study, ensuring 60 patients complete the study period with bempedoic acid. This also means that recruitment will be stopped once 60 patients have continuously taken the investigational product and the background medication of atorvastatin (at least 40 mg/day, or equivalent) plus ezetimibe (10 mg/day) until V3, with blood tests completed at all visits.

All patients admitted to the Department of Cardiology with acute coronary syndrome will be screened for eligibility.

## Statistical analysis

All analyses will be descriptive and performed in the ITT population. Demographic and baseline measurements will be summarized using descriptive methods. In the primary analysis, the proportion of responders and an exact descriptive 95% Clopper–Pearson confidence interval (CI) will be calculated. Patients with missing values for the primary endpoint will be counted as treatment failures in the ITT analysis, that is, as patients who did not achieve LDL-C guideline targets of <1.4 mmol/L (55 mg/dL) after treatment for 14 weeks.

Explanatory analyses for secondary endpoints will be carried out separately for Groups A and B. Responder rates will be examined using the exact 95% Clopper–Pearson CIs, and continuous data will be compared exploratorily using two-sided paired *t*-tests, with a two-sided level significance of 5%. In addition, mean changes in continuous laboratory parameters will be compared between Groups A and B using an analysis of covariance model, including baseline values at the start of the study and group assignment.

For the primary endpoint, a subgroup analysis will be carried out. Patients in the triple therapy group who fail to achieve the LDL-C guideline target of LDL-C < 1.4 mmol/L (55 mg/dL) after Stage I will be divided into four subgroups depending on their LDL-C level:

1. Group: LDL-C of 55 – ≤ 68.75 mg/dL (1.4–≤1.8 mmol/L).
2. Group: LDL-C of 68.75 – ≤ 82.5 mg/dL (1.8–≤2.1 mmol/L).
3. Group: LDL-C of 82.5 – ≤ 96.25 mg/dL (2.1–≤2.5 mmol/L).
4. Group: LDL-C of 96.25 – ≤ 110 mg/dL (2.5–≤2.8 mmol/L).

In every subgroup, the proportion of responders will be examined and exact 95% Clopper–Pearson CIs will be calculated. To evaluate the robustness of the results, efficacy analyses will be repeated in the PP population (sensitivity analyses).

Descriptive analyses will be performed to assess adverse events and serious adverse events. As this is an exploratory study, no adjustment for multiplicity will be performed. Confidence intervals and respective *p*-value will only be assessed descriptively.

Access to the final trial dataset will be centralized at the Center for Clinical Trials.

## Pharmacovigilance

Adverse events will be recorded for all participants from the first application of bempedoic acid (or the equivalent time point for the double therapy group) until the last study visit. The safety of add-on treatment with bempedoic acid on top of atorvastatin and ezetimibe background therapy will be assessed by descriptive comparison of (serious) adverse events between the two treatment arms. This safety assessment (as detailed in Table 1) includes but is not restricted to the repetitive measurement of uric acid and creatine kinase as well as the assessment for tendon-related or other muscle-associated potential side effects.

## Trial status

The study design described corresponds to protocol version 3.0 dated 13 November 2023. Patients were recruited between July 2023 and March 2026.

## Limitations

The study has a single-center, open-label, non-randomized design, which introduces potential bias. For example, the single-arm design inherits the risk that changes in food intake, general lifestyle, and background medication over time may influence LDL-C and, thereby, the estimate of LDL-C-lowering effects of bempedoic acid. Hence, our study is exploratory regarding the escalation strategy and non-comparative.

HACOL-ACS is a short-term feasibility trial testing the applicability of sequential add-on bempedoic acid when guideline-recommended LDL-C targets are not achieved with tolerated high-intensity statin treatment combined with ezetimibe. The purpose of the trial is to describe the extent of additive LDL-C lowering provided by the sequential escalation. Given the small sample size and short duration, the trial does not assess long-term outcomes and, therefore, relies on surrogate endpoints, limiting clinical inference.

The single-center design and the fact that the study will be conducted within a specific healthcare system with defined prescribing constraints will impact but not prevent external validity and applicability to other settings. However, these prescribing constraints would have similar applicability even in a multicenter trial in a single country. The constraints on lipid-lowering therapy, however, allowed for prescription of atorvastatin, rosuvastatin, and ezetimibe, which will allow to use the most potent statins.
